# Supplementary figures and images for: Rapid mechanosensitive migration and dispersal of newly divided mesenchymal cells aid their recruitment into dermal condensates
Source: PLoS Biol. 2023 Sep 25;21(9):e3002316. doi: 10.1371/journal.pbio.3002316 (PMC10553821; doi:10.1371/journal.pbio.3002316)

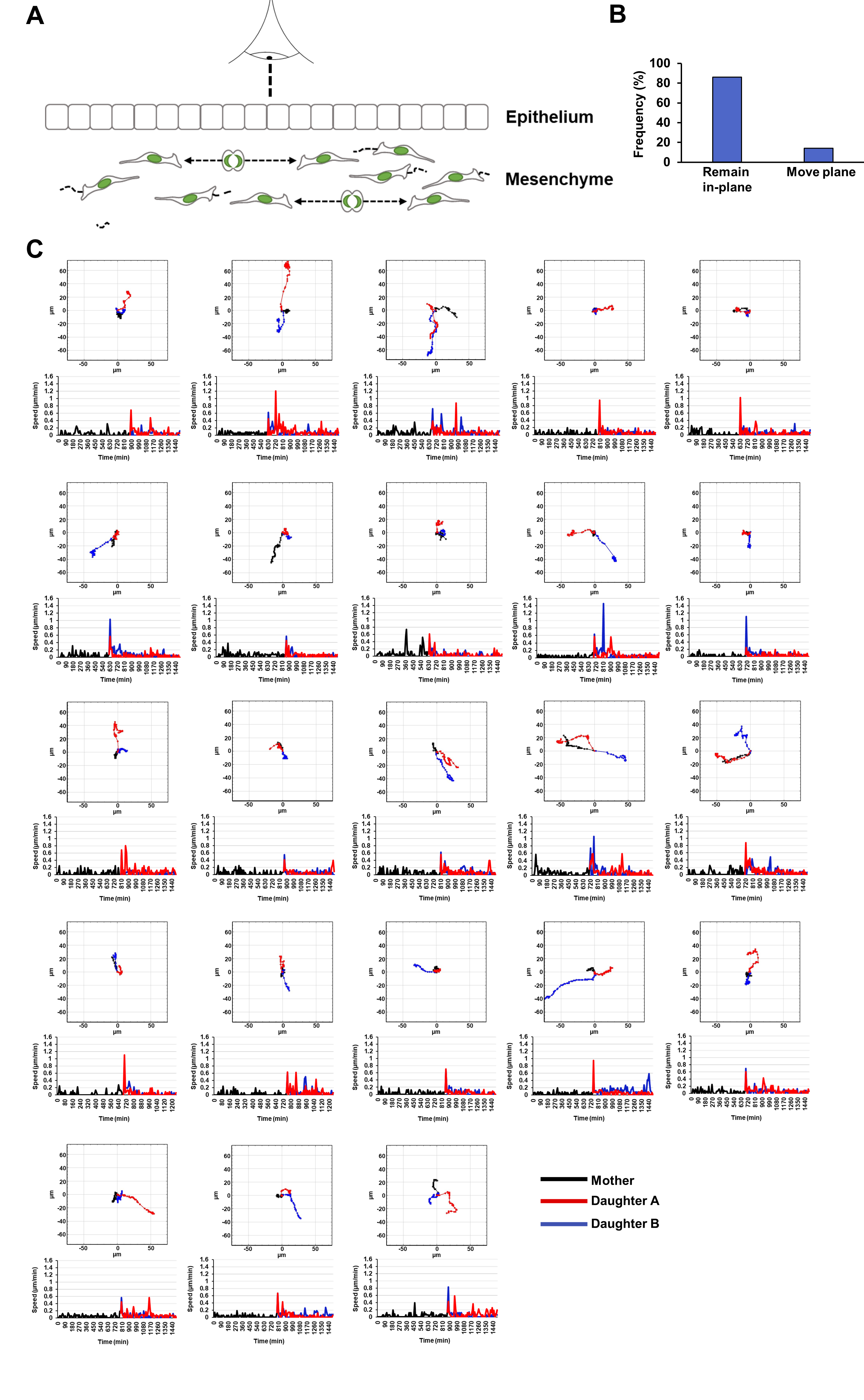

Supplement: S1 Fig — (A) Schematic depicting the position of mesenchymal cells migrating and dividing in a planar manner, parallel to the epidermis. Orientation of view is shown. (B) Quantification of cells remaining in-plane after 3 h of imaging (number of tracked daughter cells = 100; z-slice depth = 6 μm). (C) Mitosis plots mapping mother and daughter tracks before and after cell division, with corresponding cell speed plots over the time course of imaging below. Tracks were chosen from cells in which cell division took place within a time point of 40%–60% through the imaging, and both daughter cells were tracked until the end of the video. The raw numerical values and tracking data for B and C can be found in S1 Data. (TIF) [file pbio.3002316.s001.tif]

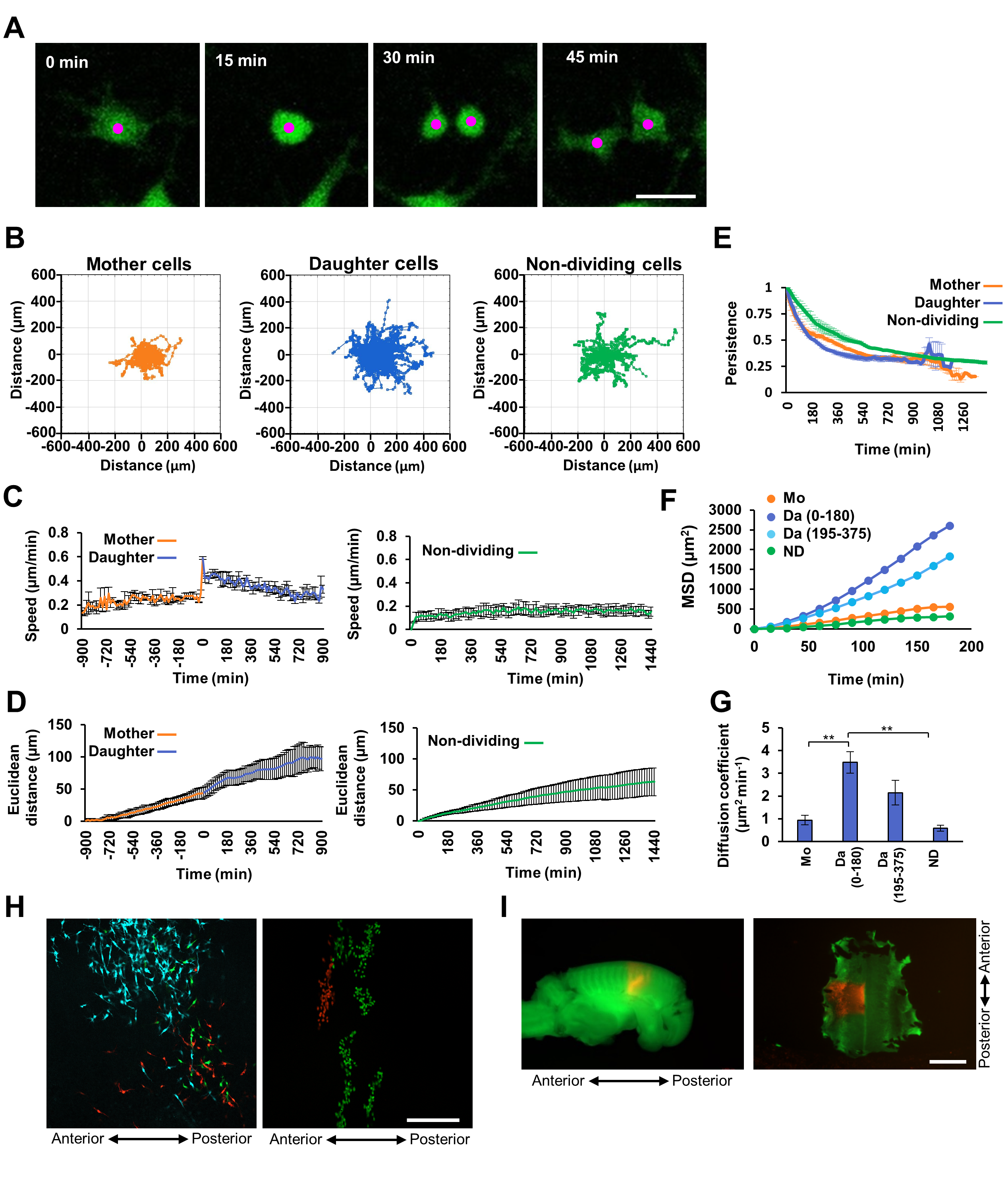

Supplement: S2 Fig — (A) Single z-planes from confocal time-lapse series of an E6 Chameleon chicken skin explant culture treated with cell permeant TAT-Cre and cultured for 24 h. Magenta dots highlight mother and daughter cells. (B) Diffusion plots showing the migration direction and dispersion of tracked mother (left; n = 62), daughter (middle; n = 124), and non-dividing (right; n = 50) cells from a single skin explant. (C, D) Speed (C) and Euclidean distance travelled (D) of dividing (left panels; time 0 = point of mitosis) and non-dividing (right panels) cells from Chameleon chicken skin explants. (E) Persistence (Euclidean/accumulated distance) of mother, daughter, and non-dividing cells against time. (F) Mean squared displacement (MSD) of mother cells (Mo; n = 62) for 180 min prior to division, daughter cells for 180 min after division, daughter cells for 180–360 min after division (Da; n = 124 in both windows), and non-dividing cells (ND; n = 50) for a single representative skin explant. (G) Mean diffusion coefficient (the slope of the line in F) of mother cells (Mo), daughter cells (Da) for 180 min after division, daughter cells for 180–360 min after division, and non-dividing cells (ND). A one-way analysis of variance (ANOVA (p < 0.01)) followed by pairwise post hoc Tukey’s honestly significant difference tests revealed a significant difference between daughters (0–180 min) and mother cells, and between daughters (0–180 min) and non-dividing cells (*p < 0.05, **p < 0.01). The raw tracking data for B–G can be found in S5 Data. (H) Confocal images of clonally labelled mesenchymal (left) and epithelial (right) cells in E7 Chameleon skin explant culture following somite and overlying epithelium injection of cell permeant TAT-Cre at E3. (I) Tissue derivatives of a tdTomato somite (TPZ transgenic line) transplanted into a CAG-GFP host embryo, at E6.5. Left panel, intact embryo; right panel, isolated skin. C, D, E, and G time-lapse videos n = 3 from 3 independent samples, mean number o [file pbio.3002316.s002.tif]

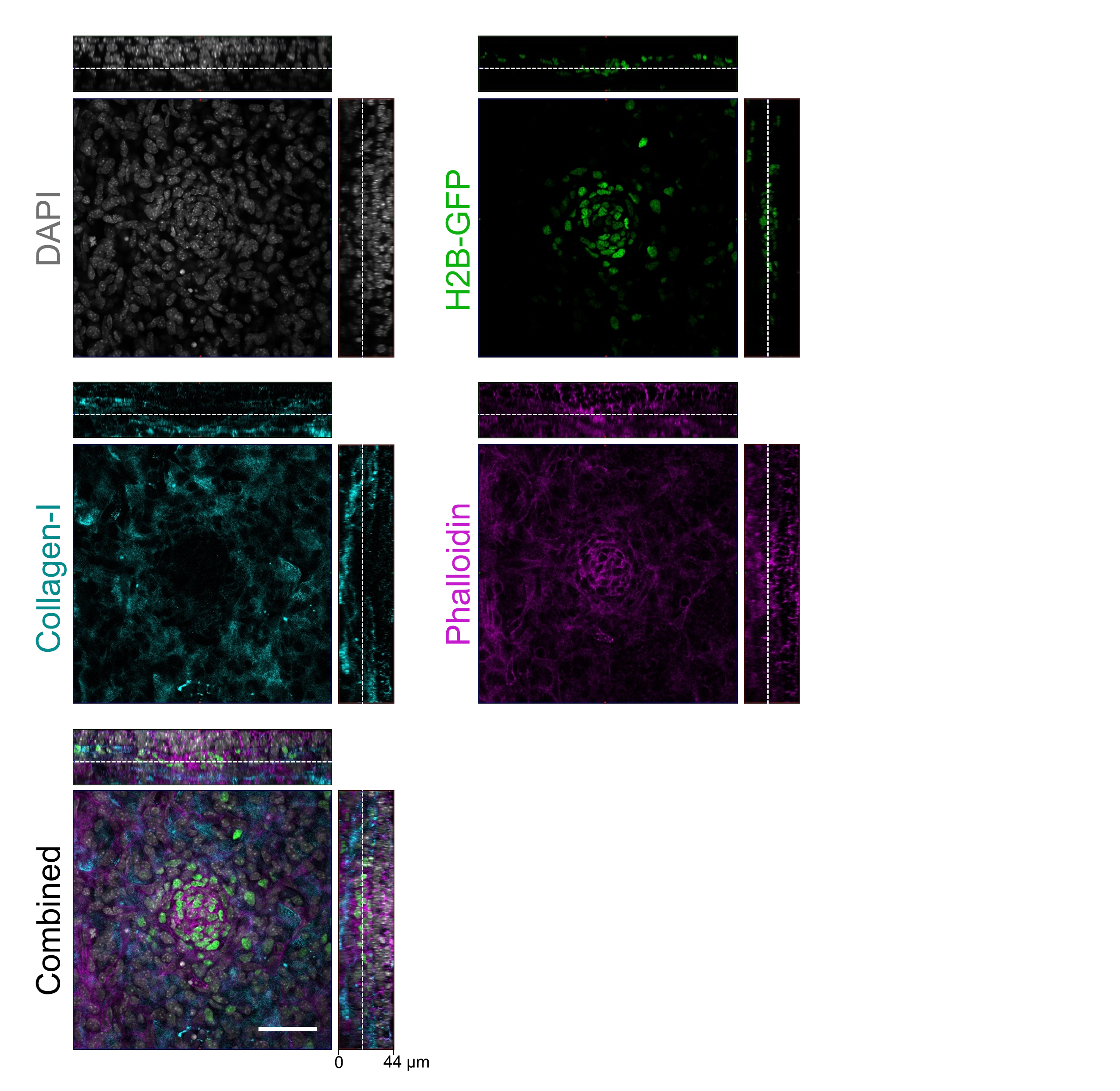

Supplement: S3 Fig — Single planes and orthoviews from confocal imaging of Collagen-I immunofluorescence in E14.5 TCF/Lef::H2B-GFP skin explant stained with phalloidin (to detect F-actin) and DAPI. White dashed lines indicate the plane of the z-section. Scale bar = 50 μm. (TIF) [file pbio.3002316.s003.tif]

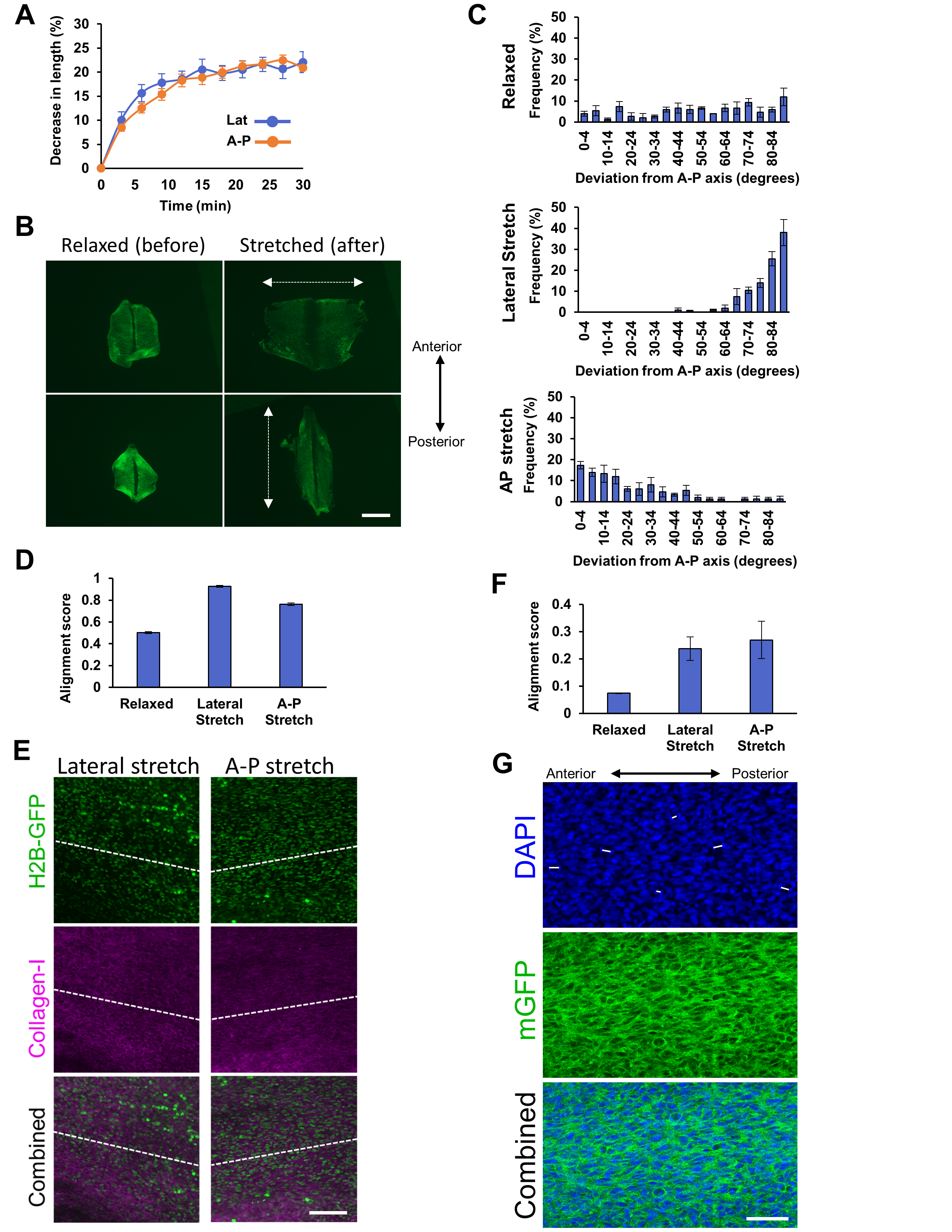

Supplement: S4 Fig — (A) Plot showing the percentage decrease in anterior-posterior (A-P) and dorsal-ventral (Lat) length of mouse skin explants in a 30 min period (n = 5) when suspended freely in culture medium. (B) Images of E13.5 TCF/Lef::H2B-GFP mouse skin explants before and after a lateral stretch (upper panels) or a stretch along the anterior-posterior (A-P) axis of the embryo (lower panels). White dashed arrows show direction of stretch. (C) Nucleus orientation angle for relaxed (n = 3; upper), lateral stretched (n = 4; middle), and A-P stretched (n = 3; lower) skins. (D) Alignment score of nucleus orientation angle from relaxed (n = 3), laterally stretched (n = 4), and A-P stretched (n = 3) skins. (E) Single planes from confocal imaging of Collagen-I immunofluorescence in E13.5 TCF/Lef::H2B-GFP skin explants in stretched states. Dashed white line indicates direction of stretch. (F) Alignment scores of Collagen fibres from skin samples shown in E. The raw numerical values for A, C, D, and F can be found in S4 Data. (G) Single planes from confocal imaging of an A-P stretched E7 membrane GFP (mGFP) chicken skin. Daughter nucleus pairs are connected by white lines, showing coherent angles of mitosis aligned with applied tension. Error bars represent SEM. Scale bar in B = 2 mm; scale bar in E = 100 μm; scale bar in G = 50 μm. (TIF) [file pbio.3002316.s004.tif]

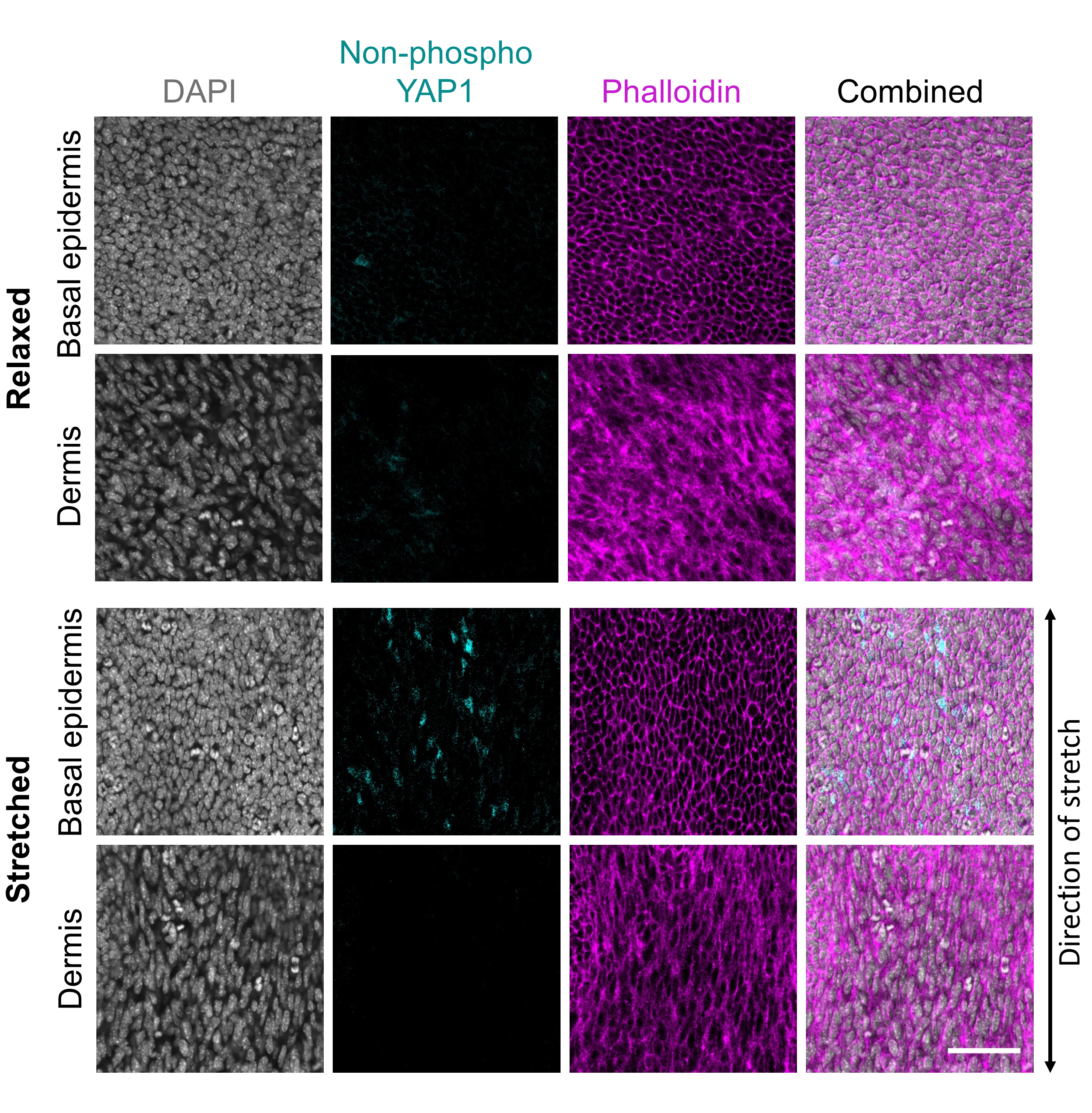

Supplement: S5 Fig — Single planes of the dermis and basal epidermis from confocal imaging of active (non-phospho) YAP1 immunofluorescence in E13.5 mouse skin explants, stained with phalloidin (to detect F-actin) and DAPI, in relaxed and laterally stretched states. Scale bar = 50 μm. (TIF) [file pbio.3002316.s005.tif]

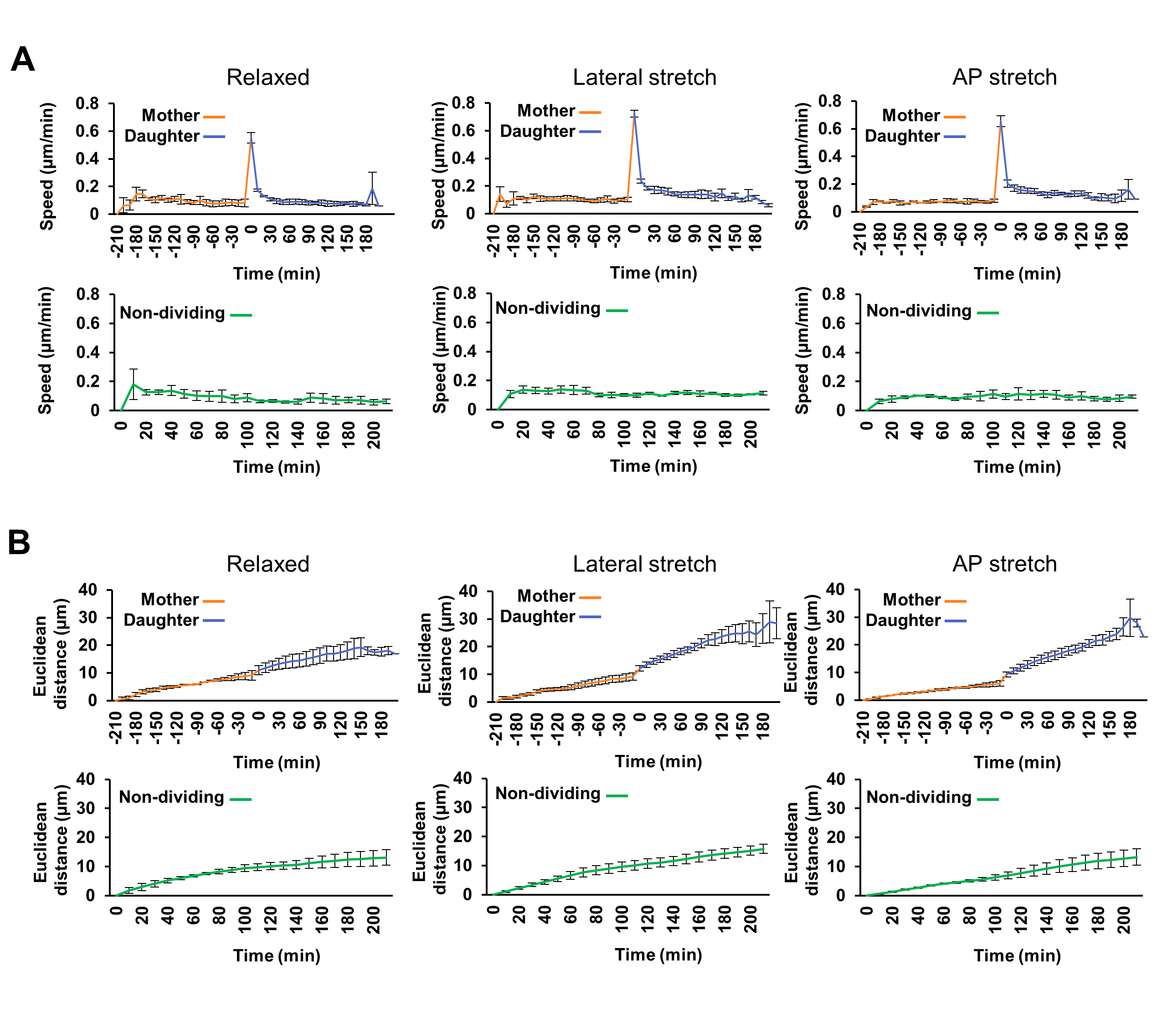

Supplement: S6 Fig — (A, B) Speed (A) and Euclidean distance travelled (B) of tracked dividing (top panels; time 0 = point of mitosis) and non-dividing (bottom panels) cells in skins that were relaxed (n = 3, average number of dividing cells tracked/video = 67, non-dividing cells tracked/video = 50), stretched laterally, (n = 4, average number of dividing cells tracked/video = 72, non-dividing cells tracked/video = 50), and stretched along the A-P axis (n = 3, average number of dividing cells tracked/video = 79, non-dividing cells tracked/video = 50). The raw tracking data for A and B can be found in S4 Data. Error bars represent SEM. (TIF) [file pbio.3002316.s006.tif]

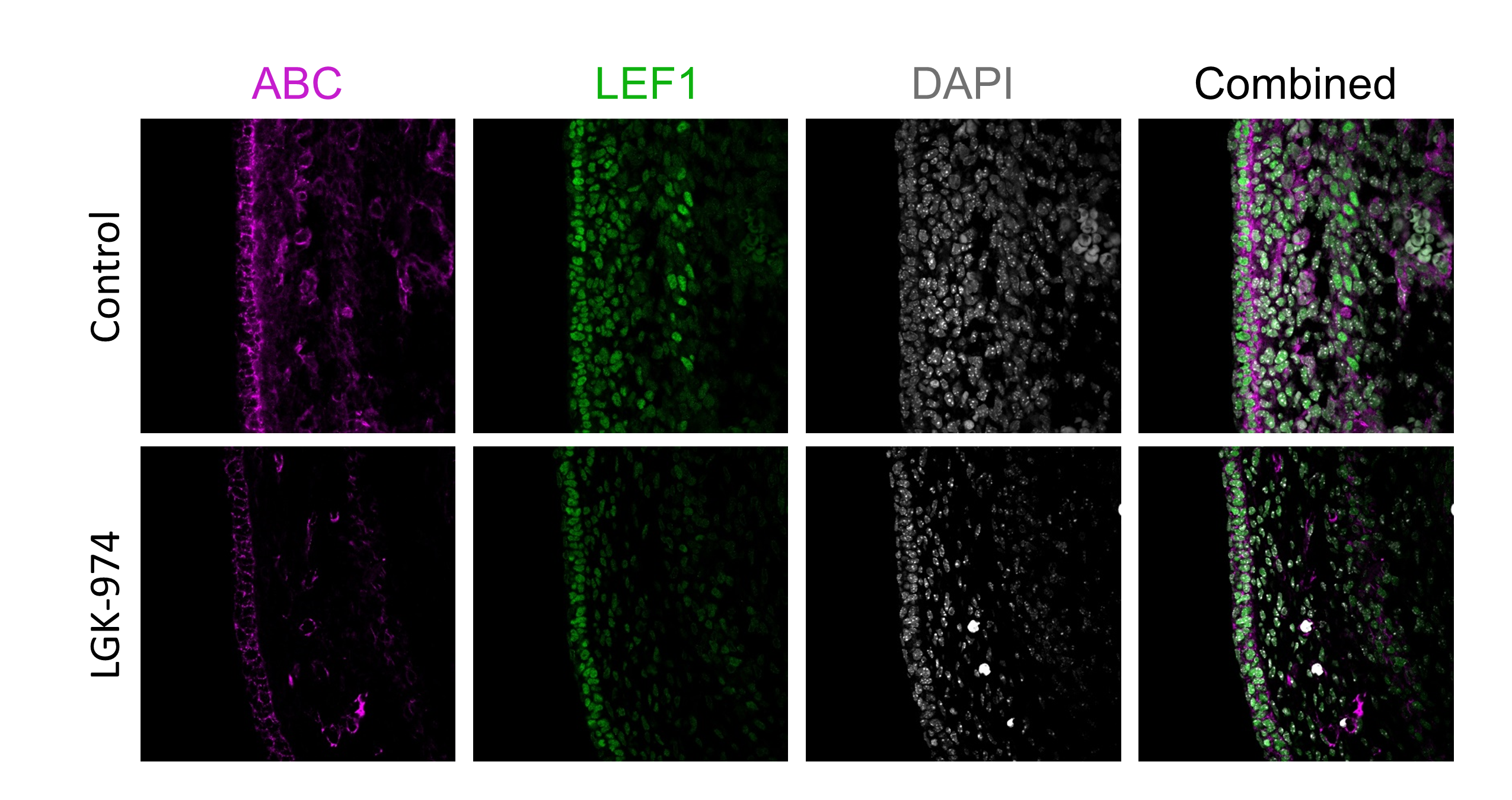

Supplement: S7 Fig — Confocal imaging of active β-catenin (ABC) and LEF1 immunofluorescence in E13.5 TCF/Lef::H2B-GFP skin sections, stained with DAPI, from embryos from pregnant mice untreated (control) or treated with LGK-974 for 24 h. Less active β-catenin signal is detected in the mesenchyme of treated embryos. Scale bar = 50 μm. (TIF) [file pbio.3002316.s007.tif]

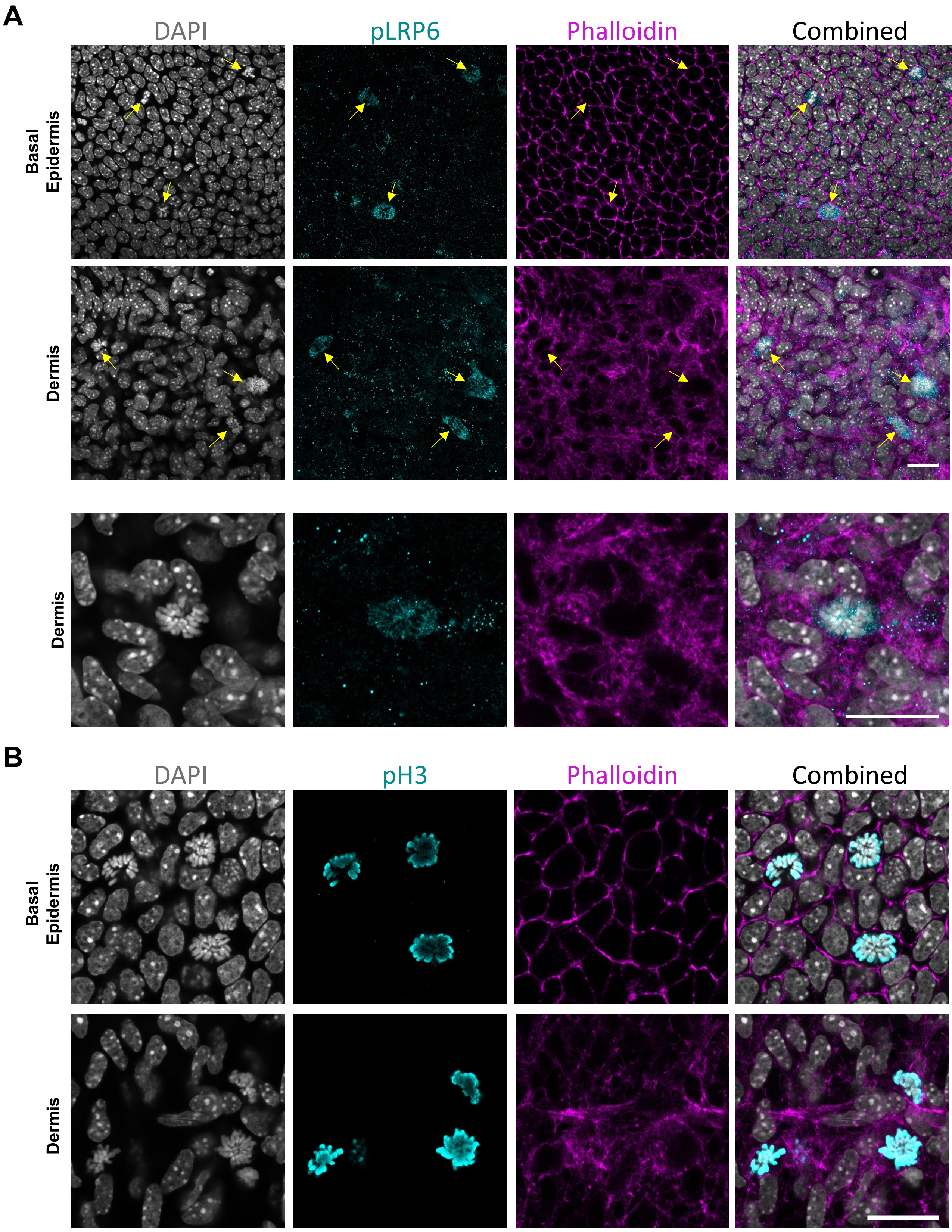

Supplement: S8 Fig — (A, B) Single planes of the dermis and basal epidermis from confocal imaging of phospho-LRP6 (S1490) (A) and phospho-histone H3 (S10) (B) immunofluorescence in E13.5 mouse skin explants, stained with phalloidin (detecting F-actin) and DAPI. Phospho-histone H3 signal illustrates the morphology of nuclei undergoing mitosis. Yellow arrows indicate cells that are in mitosis and have condensed chromatin. Phospho-LRP6 (S1490) is detected at highest levels in cells undergoing division in mesenchyme and epithelium. Scale bar = 20 μm. (TIF) [file pbio.3002316.s008.tif]
