# Supplementary material for: Rapid mechanosensitive migration and dispersal of newly divided mesenchymal cells aid their recruitment into dermal condensates
Source: PLoS Biol. 2023 Sep 25;21(9):e3002316. doi: 10.1371/journal.pbio.3002316 (PMC10553821; doi:10.1371/journal.pbio.3002316)
Supplement: Attachment — Submitted filename: Riddel et al critique 2023.docx [file pbio.3002316.s025.docx]

Riddel et al., Critique 2023 Plos Biology

The manuscript, authored by Riddell et al., is a characterization of dermal fibroblasts progenitors that contribute to the dermal condensate in mouse and chicken embryonic skin. Their observations show a rapid, persistent, migration in a three-hour period. They found that (a) the newly born dermal mesenchymal cells migrate rapidly and persistently resulting in greater displacement upon and following mitosis compared to the non-dividing cells in mouse embryos. (b) The newly born mesenchymal cells are more likely to encounter the dermal condensates. (c) The orientations of mitoses are aligned in the mouse embryonic mesenchyme cultured *ex vivo* while the global mesenchymal cell division angles are not aligned in the intact E13.5 embryos, suggesting the mitotic orientation is possibly influenced by the topology of tissue. (d) The orientation of the newly born mesenchymal cells are sensitive to the orientation of tension.

Their findings are interesting, relevant, and provocative. They have elegant imaging techniques to visualize the cell behaviors in the dermis. The major concern with this report is the study design is largely descriptive in wildtype over a short time period predominantly and one aspect such as tension is manipulated in figure4. The authors do not demonstrate which of the variables they have identified is causative for the cell behavior (orientation, collagen pattern, condensate, movement). For instance can they compare a skin model that has condensates versus not in chick or mouse? Or can they use pharmacological inhibition of collagen assembly? Or can they manipulate a specific aspect of mechanosensation (stretch is complex because it can also affect planar cell polarity) ? One of the main findings such as “recruitment” of daughter cells into the condensates concept is not clearly demonstrated in their results or experimental strategies. Couldn’t the spreading migration makes just make it more plausible and convenient for daughter cells to encounter the condensate?

Here are some additional concerns and suggestions:

Introduction text: Introduce some more relevant detail and context for the early events in pre-dermal condensate morphogenesis at the cell biology level. Recent review from Myung P. et al., in *Development* 2022 on this issue might be cited. Why mention dermal adipocyte origin because it is not relevant to this study. What does it mean that there is “high developmental potential” of fibroblasts?

**For Figure 1:**

**Figure 1K**: Microenvironment analysis is cursory. There can be more differences than just collagen. It does not add to the analysis of the wild-type scenario. There is not that much mature collagen protein. Which type of collagen? In this window there is not that much collagen protein.

Figure1 will be easier to understand if it could include a simple schematic depicting the position of the ex vivo mouse skin tissue and the mitotic dermal cells they are testing and the displacement direction of those mesenchymal cells. In **Fig 1C**, it turns out that the migration speed of the non-dividing cells is close to 0, however the **Fig 1D** indicates that the non-dividing cells still showed increasing Euclidean distance during the time period. And in text, they indicate that the daughter cells result in ‘a mean Euclidean displacement ~50% greater than’ the non-dividing cells, but it is not displayed in **Fig 1G**.

For **Fig 1J** and **S7 video**, authors suggested they observed some mitoses occurring in dermal condensates while cells in dermal condensates are largely quiescent. What is the incidence of mitosis in dermal condensates under their observation? Have they also observed similar mitosis in chicken embryonic skin mesenchymal cells?

**Figure 2:**

In text, they suggested that they found the orientation of mitosis is at a tangent to the condensate perimeter and the collagen ring is generated surrounding the condensate, thus they concluded that the extracellular environment surrounding the dermal condensate influences the orientation of cell migration and mitosis. This is not shown with causative data or ruling out other possibilities. But what if the orientations of the cell movement and the mitosis are already set up in a chemotaxis manner for entering and constituting the dermal condensate later? To rule out this possibility, it would be convincing to show the expression of signals such as FGF20 and SHH, or show the cell polarity distribution a day earlier.

**Figure 2 text:** Does the original orientation of division relative to condensate influences the direction of displacement of the daughter cells? If these cells are not oriented directly towards the condensate (lines 173-176) to begin with, how do they get displaced towards the condensate? **Figure 2D**: If dermal condensates influence division angle within this radius, can they show this in a causative manner in a system that lacks dermal condensates in chick or mouse.

**Figure 2D**: Similarly, if collagen ring surrounding the condensate “influences the orientation of the cells and direction of mitosis” can they show this in a collagen mutant or Loxl2-inhibition models. Which collagen (collagen 1 or 3)? Also, the image is not clearly annotated for peri-condensate nuclei alignment.

**Figure 3:**

In **figure 3**, the in vivo imaging shows regional alignment of mitotic cells. Where is this in relation to the condensate? If it is regionally aligned, how large are the regions.

**Figure 2C & 3F:** Adding the coordinate system that the angles come from would make it slightly quicker to interpret. Like where is 0 degrees?

**Figure 4:**

For **Fig 4G**, how to explain that the non-dividing cells in relaxed tissue show a higher speed than them in stretched tissue in the first 20 mins?

**Lines 289 and 290:** What makes newly born mesenchymal cells enter a more mechanosensitive state in early G1? It is interesting that the mother cells do not demonstrate the same speed of movement but could they contribute to the mechanism at all of what is going on during G1 to make newly born cells more sensitive?
