# Supplementary material for: Rapid mechanosensitive migration and dispersal of newly divided mesenchymal cells aid their recruitment into dermal condensates
Source: PLoS Biol. 2023 Sep 25;21(9):e3002316. doi: 10.1371/journal.pbio.3002316 (PMC10553821; doi:10.1371/journal.pbio.3002316)
Supplement: Attachment — Submitted filename: Response to Reviewers Riddell et al..docx [file pbio.3002316.s026.docx]

**Response to Reviewers Requests -** PBIOLOGY-D-23-00306R2 Riddell et al.

**Reviewer remarks:**


**Reviewer #1:** The authors of Riddell et al. have been largely able to respond to the criticism I raised, either by deleting data, providing new data and/or new analysis/images. In S3, I would appreciate seeing the plane of the z-section in the orthoviews to still better appreciate at what depth the optical section is from.
The new data on the role of the Wnt pathway is intriguing, having seen the effect on the migration of newly born cells (as in Fig. 1) without strecth would have interesting.

**Response:** We have added a dashed white line to the orthoviews in Figure S3 to indicate the depth of the z-section shown, as requested.


**Reviewer #2:** Thank you for the extensive revisions on the text and figures and the inclusion of some functional experiments.  The focus on the Wnt pathway is a good addition to the paper and demonstrates the pre-DC events. Your findings are consistent with the scRNA-seq and functional analysis from the Myung and Rendl labs.  I apologize for the delay.  I did not get the reviewer responses with the manuscript files.

**Response:** We thank the reviewer for their comments.


**Reviewer #3:** In the revised manuscript, Riddell et al. have strengthened this interesting study. Notably, they have shown that the pharmacological inhibition of Wnt by LGK-974 disrupts the alignment of mitosis along the stretch and reduces the migratory speed of the newly born daughter cells. Furthermore, they have demonstrated that LRP6 phosphorylation peaks at mitosis, providing a plausible mechanism for enhanced Wnt signaling at mitosis, which subsequently affects daughter cell migration. Therefore, I have no further concerns about the manuscript. However, the Wnt-dependent behaviors of cell division and post-division migration are highly interesting. It is known that basal epithelial cells are the major source of WNT ligand production during embryonic development, and this generates a gradient of WNT signaling from the epidermis to the dermis. The authors should discuss whether they observed a differential migratory speed in newly born mesenchymal daughter cells, depending on the distance of these cells from the basal epidermis, and whether WNT high mesenchymal cells (marked by TCF:LEF-H2bGFP reporter) migrate more rapidly in general.

**Response:** We did not observe an immediately apparent difference in migratory speeds at different distances from the epidermis. Whether or not this occurs would depend on whether the WNT effect on migration of newly born mesenchymal cells is dose-dependent, or binary. We have noted the importance of this issue to be addressed in future work to the discussion (lines 343-5).
